# Semaglutide ameliorates metabolism and hepatic outcomes in an NAFLD mouse model

**DOI:** 10.3389/fendo.2022.1046130

**Published:** 2022-12-09

**Authors:** Shu Niu, Shuchun Chen, Xing Chen, Qingjuan Ren, Lin Yue, Xiaoyu Pan, Huiying Zhao, Zelin Li, Xiaoyi Chen

**Affiliations:** ^1^ Department of Internal Medicine, Hebei Medical University, Shijiazhuang, Hebei, China; ^2^ Department of Endocrine, Shijiazhuang People’s Hospital, Shijiazhuang, Hebei, China; ^3^ Department of Internal Medical, Hebei General Hospital, Shijiazhuang, Hebei, China; ^4^ Department of Endocrine, The Third Hospital of Shijiazhuang, Shijiazhuang, Hebei, China

**Keywords:** semaglutide, hepatic, NAFLD, hepatic fibrosis, metabolomics

## Abstract

**Purpose:**

The aim of this study was to evaluate changes in body weight, liver weight, blood glucose, liver injury markers, pro-inflammatory factors and oxidative stress marker levels in obese mice with HFD induced NAFLD after semaglutide use.

**Patients and methods:**

The 24 C57BL6J mice were randomly divided into three groups (NCD, HFD and Sema) for the assessment of metabolic status, inflammatory factor and oxidative stress marker levels, liver histopathology in mice. Liver metabolomics was determined by liquid chromatography/mass spectrometry (LC-MS) method.

**Results:**

The mice body weight, liver weight, blood glucose, TG, TCHO, LDL and pro-inflammatory factors were significantly reduced after semaglutide. Meanwhile, semaglutide increased the SOD level. Semaglutide treatment significantly improved the pathological changes such as hepatocyte steatosis, balloon degeneration and lymphoid foci by HE. It also significantly reduced lipid droplet by Oil Red O. The mitochondria were swollen, the volume increased, the cristae were partially broken and reduced, the intramembrane matrix was partially dissolved, and the mitophagy structure was visible in the visual field. There were 6 metabolites down-regulated and 2 metabolites significantly up-regulated after semaglutide treatment.

**Conclusions:**

Semaglutide can reduce blood glucose level and liver fat accumulation and play an anti-inflammatory role in advanced NAFLD that due to the effects of HFD.

## Introduction

Nonalcoholic fatty liver disease (NAFLD) is becoming an increasingly prevalent form of chronic liver disease in the Western world. NAFLD is a multifactorial clinicopathological syndrome characterized by hepatocyte lipid accumulation and hepatocyte steatosis, including simple fatty liver and nonalcoholic steatohepatitis (NASH). NAFLD can progress from simple steatosis (i.e., nonalcoholic fatty liver [NAFL]) to NASH, cirrhosis, and cancer. In addition to steatosis, NASH is characterized by inflammation, ballooning degeneration, and fibrosis, which ultimately leads to liver fibrosis and end-stage cirrhosis (1). Hepatocellular carcinoma in the setting of non-cirrhotic nonalcoholic fatty liver disease and the metabolic syndrome: The global incidence of NAFLD is 6-35% (median 20%) (2, 3). Interestingly, NAFLD with hepatic fibrosis is strongly associated with hepatic and cardiovascular mortality (4, 5). It is well known that diet control combined with physical exercise can reduce the occurrence of fatty liver, especially diet control. However, dietary changes do not treat established NAFLD. GLP-1 receptor agonists have been shown to be effective in the treatment of NAFLD patients with diabetes (6), but there is lack of evidence for its efficacy and mechanism of action in non-diabetic NAFLD patients (7). As a GLP-1 receptor agonist, semaglutide has good clinical efficacy in the treatment of NAFLD with diabetes (8). The purpose was to investigate whether semaglutide has anti-inflammatory and anti-fibrotic effects on non-diabetic NAFLD while improving hepatic steatosis. Therefore, we constructed an obesity model with advanced NAFLD without diabetes assessed by liver histology. To further explore the potential mechanism of semaglutide intervention in NAFLD. Metabolomics mainly studies the metabolic pathways of small molecules and their metabolites. At present, metabolomics technology has been widely used in the research of NAFLD drug treatment, such as: drug effect evaluation, drug screening, and drug action mechanism research (9). This study was the first to investigate the effect of semaglutide on liver metabolomics. The hepatic metabolomic profile was determined by liquid chromatography/mass spectrometry (LC-MS) method to explore the regulatory targets and underlying mechanisms of semaglutide on non-diabetic NAFLD.

## Materials and methods

### Animal model

Male C57BL/6J mice (6 weeks old) were purchased from Hebei Ivivo Biotechnology Co., Ltd. (Shijiazhuang, China). All mice fed in specific-pathogen free (SPF)-room under the 12h-alternation cycle of day/night, temperature of (24 ± 2) °C, and relative humidity of (60 ± 10) %. Following one week of acclimation, mice were randomly divided into normal diet group (4% fat, 20% carbohydrate, and 20% protein, NCD group, n=9) and high-fat diet (60% fat, 20% carbohydrate, and 20% protein, HFD group, n=18). After 12 weeks of feeding, mice in the HFD group were subdivided into high-fat diet control group (HFD, n=9) and semaglutide intervention group (HFD+sema, n=9). Specifically, mice in the HFD+sema group were intraperitoneally injected with 30 μmol/kg semaglutide (Novo Nordisk, Copenhagen, Denmark) once daily for another 12 weeks. The highest dose of semaglub was selected on the basis of previously published studies in mice (10). The mice in the NFD and HFD groups received an equal of phosphate-buffered saline (PBS). Mice were anesthetized by intraperitoneal injection of pentobarbital sodium (40mg/kg) and sacrificed on the 24^th^ week. After that, blood samples and liver tissues were collected and stored for further analyses.

### Intraperitoneal glucose tolerance test

Intraperitoneal glucose tolerance test (IPGTT) was conducted referring to previous study (11). Specifically, mice were fasted overnight, followed by intraperitoneal injection of glucose (2 g/kg). Blood samples were collected at 0, 15, 30, 60, 90, and 120 min and measured by glucometer (German Roche Rokan total glucose meter).

### Biochemical analysis

Blood was collected and then centrifuged to obtain the serum. Serum levels of alanine transaminase (ALT), aspartate transaminase (AST), triglyceride (TG), total cholesterol (TC), low-density lipoprotein (LDL) and high-density lipoprotein (HDL), and were measured using corresponding kits (Nanjing Jiancheng, Nanjing, China).

### Measurement of inflammatory cytokines and oxidative stress marker

The concentrations of inflammatory cytokines including tumor necrosis factor-α (TNF-α), interleukin (IL)-6, and IL-1β, and oxidative stress marker including malonaldehyde (MDA) and superoxide dismutase (SOD) were detected by ELISA kits (Lianke, Hangzhou, China).

### HE staining

Liver tissues were fixed with 4% paraformaldehyde, embedded in paraffin, and sliced into 5 µm sections. Afterwards, the sections were dewaxed, rehydrated, and stained with hematoxylin-eosin (HE). Images were captured by microscope (ECLIPSE Ci-L, Nikon, Tokyo, Japan).

### Oil red O staining

Liver tissues were frozen followed by cutting into 10 μm thick. According to manufacture instruction, the sections were stained by Oil red O for lipid deposition evaluation. Finally, the area of lipid deposition was quantified with Image-pro plus 6.0 software (Media Cybermetrics, Rockville, MD, USA), thereby calculating the percentage of area of lipid deposition.

### Masson staining

To observe the extent of liver fibrosis, MASSON staining kit (G1006-100ml, CR2202127, Servicebio) was employed in the study. Briefly, 4 μm slices were dehydrated with a series of ethanol concentration, and then dyed with Wiegert’s iron haematoxylin solution. After staining with Lichun red acidic magenta solution, slices were differentiated with phosphomolybdic acid, dyed with aniline blue dye solution, and rinsed with glacial acetic acid. Images were captured by microscopy (ECLIPSE Ci-L, Nikon, Tokyo, Japan).

### Transmission electron microscopy

Liver tissues were dissected from mice and then immediately fixed in 2.5% glutaraldehyde at 4°C. After washing, tissues were post-fixed in 2% osmium tetroxide, dehydrated in ethanol/propylene oxide and embedded in epoxy resin. Then, liver tissues were cut at 70 nm thickness using ultramicrotome (Leica UC7, Leica), and next stained with uranyl acetate and lead solution. Until the sections were dry, images were captured by transmission electron microscope (HT7700, Hitachi, Tokyo, Japan), and the number of autophagosomes and autophagolysosomes was mounted by Image-proplus 6.0 (Media Cybernetics, Rockville, MD, USA).

### Liver metabolic profiling

Liver samples containing internal standard were prepared, ground, and extracted with ice bath ultrasound, followed by centrifugation. The extracts were put into liquid chromatography-mass spectrometry (LC-MS) vial, dried under nitrogen, and re-dissolved in the combination of methanol and water. Afterwards, samples were silenced at -20 °C for 2 h and centrifuged to obtain the supernatant. Supernatant was filtrated and collected for analysis using LC-MS platform. Metabolomics data were preprocessed using the Progenesis QI v2.3 (Waters Corp., Milford, MA).

### Analysis of metabolic data

Multivariate statistical analysis, such as principal component analysis (PCA), partial least squares discriminant analysis (PLS-DA) and orthogonal partial least squares discriminant analysis (OPLS-DA), was carried out after normalization of the preprocessed data matrix. Univariate analysis mainly focused on univariate description and statistical inference, and Student’s test and Fold change (FC) analysis was used to compare metabolites between two groups. The screening criteria for differential metabolites were variable important in projection (VIP) of OPLS-DA greater than 1, and p-value of Student’s test less than 0.05 or FC greater than 1, and P-value less than 0.05.

### Metabolic pathway analysis

Kyoto Encyclopedia of Genes and Genomes (KEGG) enrichment analysis was performed for differential metabolites. If the influence value of the pathway was higher than 0.05 and the P-value was less than 0.05, the pathway was considered to be significantly correlated. Bubble mapping were employed to display the metabolic enrichment pathway.

### Statistical analysis

Data were expressed as mean ± standard deviation (SD) and analyzed using the SPSS 23.0. The differences amongst different groups were compared with one-way ANOVA followed by LSD test. Statistically significance was accepted at P<0.05 level (*),P<0.01 level (**), P<0.001 level (***), P>0.05 level (ns).

### Ethic approval statement

The animal use protocol for this study has been reviewed and approved by the Animal Ethics Committee of Hebei General Hospital. The experiment was implemented in accordance with the experimental protocol, strictly regulated the operating procedures, and did not violate the ethical requirements.

## Results

### Semaglutide ameliorated HFD-induced obesity and blood glucose

**Figure 1:**
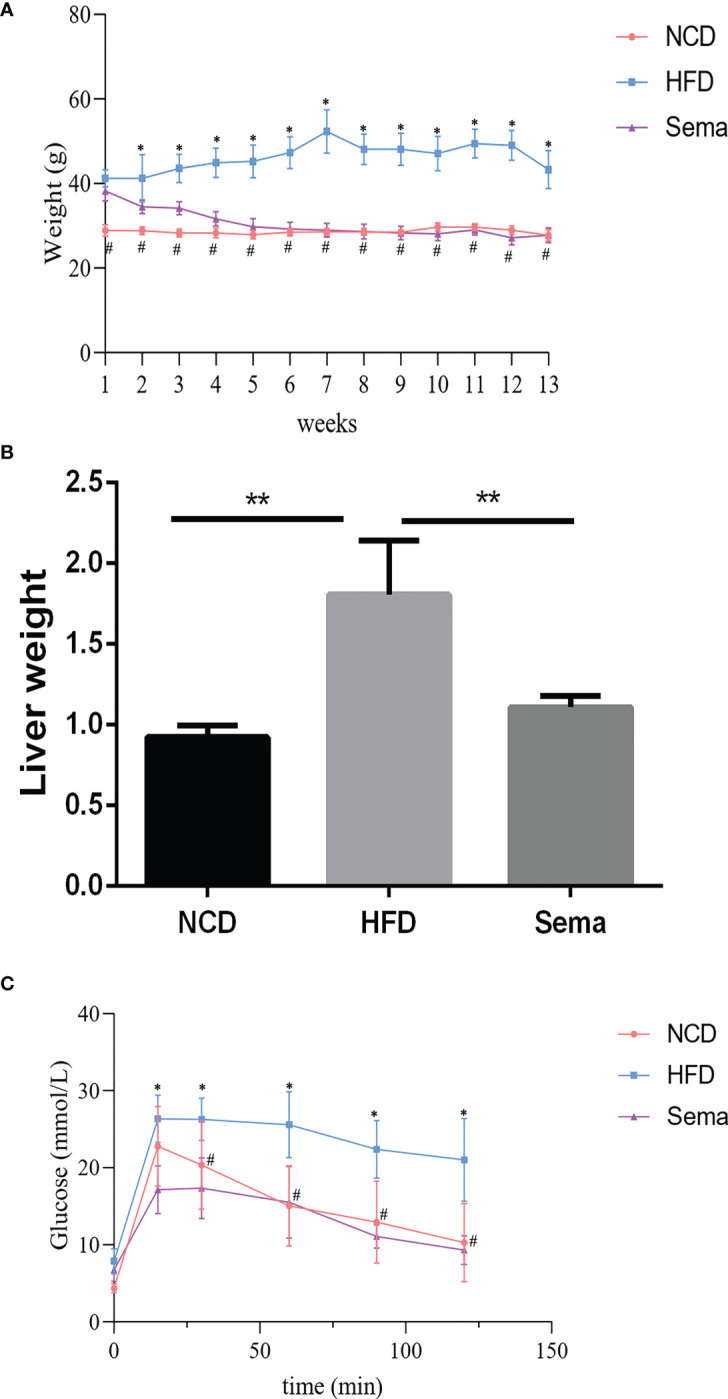
Semaglutide ameliorated HFD-induced obesity and blood glucose. **(A)** The body weight of NFD, HFD, HFD+sema group at the end of the 12th week of treatment was shown; **(B)** The liver weight of NCD, HFD, HFD+sema group at the end of the 12th week of treatment was shown; **(C)** Glucose tolerance was determined by IFGTT test; NCD, normal diet; HFD, high-fat diet; sema, semaglutide; IPGTT, Intraperitoneal glucose tolerance test. # means HFD vs Sema; * means NCD vs HFD.

To examine the function of semaglutide on NAFLD, C57BL/6J mice were fed with HFD to establish the NAFLD model *in vivo*. Results displayed that body weight in the HFD group was increased rapidly and significantly changed after the first week of feeding, compared with NCD group (P<0.05). However, the body weight was significantly reduced in the first week after administration of semaglutide (P<0.05). Additionally, the body weight of HFD+Sema group was close to that of the NCD group at the end of the 12 th week of treatment (**Figure 1A**). Correspondingly, liver weight in the HFD group were outstandingly elevated, in contrast to NCD group (P<0.05), but were declined in the HFD+Sema group (P<0.05) (**Figure 1B**). IFGTT test indicated that blood glucose was notably reduced at 15 minutes following intervention with semaglutide (P<0.05). At 60 minutes, the blood glucose level of the HFD+sema group was close to the blood glucose level of the NCD group (**Figure 1C**). Collectively, aforementioned findings clarified that semaglutide can effectively reduce the blood glucose and body weight in HFD-induced NAFLD model.

### Semaglutide alleviated the blood lipid profile and liver damage in HFD-induced NAFLD model

**Figure 2:**
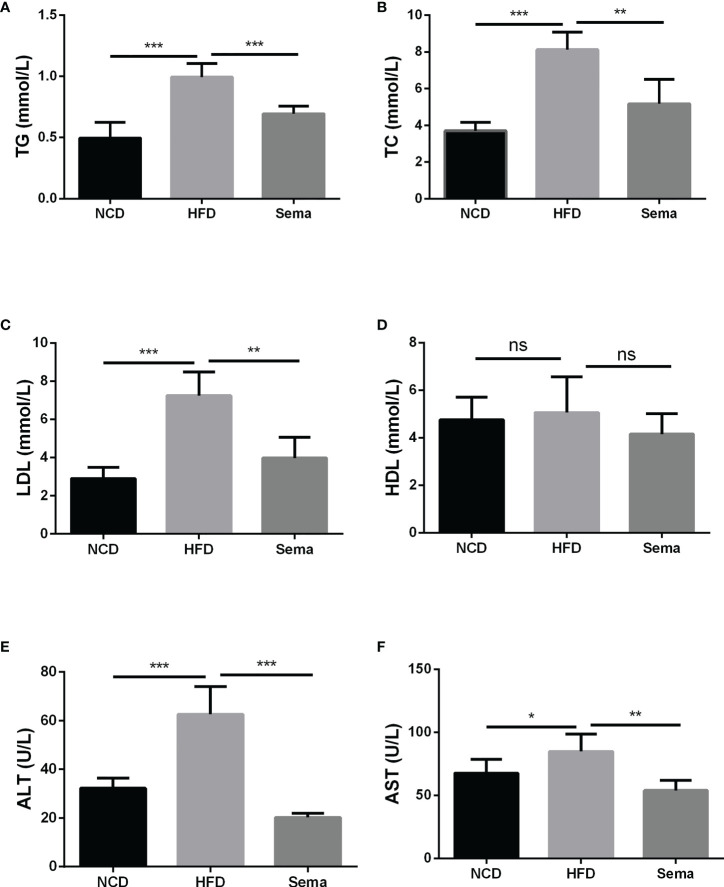
Semaglutide alleviated the blood lipid profile and liver damage in HFD-induced NAFLD model. The levels of TG **(A)**, TC **(B)**, LDL **(C)**, and HDL **(D)** were shown; The contents of ALT **(E)** and AST **(F)** in serum were shown; TG, triglyceride; TC, total cholesterol; LDL, low-density lipoprotein; HDL, high-density lipoprotein; ALT, alanine transaminase; AST, aspartate transaminase; HFD, high-fat diet; NAFLD, non-alcoholic fatty liver disease.

At the end of 24 weeks of feeding, the levels of TG, TC and LDL in HFD group were notably increased in contrast to NCD group (P<0.05). After administration of semaglutide, the contents of TG, TC and LDL were markedly down-regulated (P<0.05). However, there was no significant difference in HDL level among the different groups (P>0.05) (**Figures 2A–D**). Besides, semaglutide relieved the liver damage, as evidenced by the reduction of ALT and AST level in serum (P<0.05) (**Figures 2E, F**). Thus, we believed that semaglutide can relieve dyslipidemia and liver injury to a certain degree.

### Semaglutide reduced inflammation and oxidative stress response in HFD-induced NAFLD model

**Figure 3:**
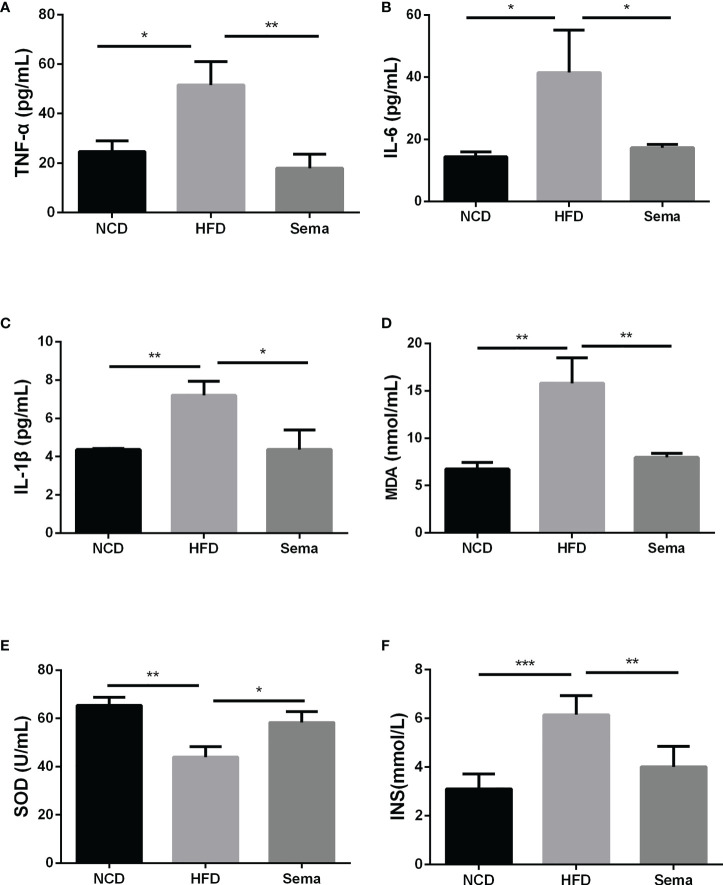
Semaglutide reduced inflammation and oxidative stress response in HFD-induced NAFLD model. The contents of TNF-α **(A)**, IL-6 **(B)**, IL-1β **(C)** and insulin **(F)** were determined by ELISA assay; The levels of MDA **(D)** and SOD **(E)** were shown; TNF-α, tumor necrosis factor-α; IL-6, interleukin-6; IL-1β, interleukin-1β; MDA, malonaldehyde; SOD, superoxide dismutase; HFD, high-fat diet; NAFLD, non-alcoholic fatty liver disease, INS, insulin.

The contents of TNF-α (**Figure 3A**), IL-6 (**Figure 3B**), IL-1β (**Figure 3C**)and insulin (**Figure 3F**)in the HFD group were higher than those of NCD group (P<0.05). By comparison with HFD group, these levels in the HFD+sema were remarkably decreased (P<0.05). Additionally, we also observed that HFD accelerated the oxidative stress response, as demonstrated by the reduction of MDA expression and elevation of SOD expression (P<0.05) (**Figures 3D, E**). Those data indicated that semaglutide may improve NAFLD through mechanisms of anti-inflammatory and anti-oxidative stress.

### Effects of semaglutide on liver histology changes in NAFLD model

**Figure 4:**
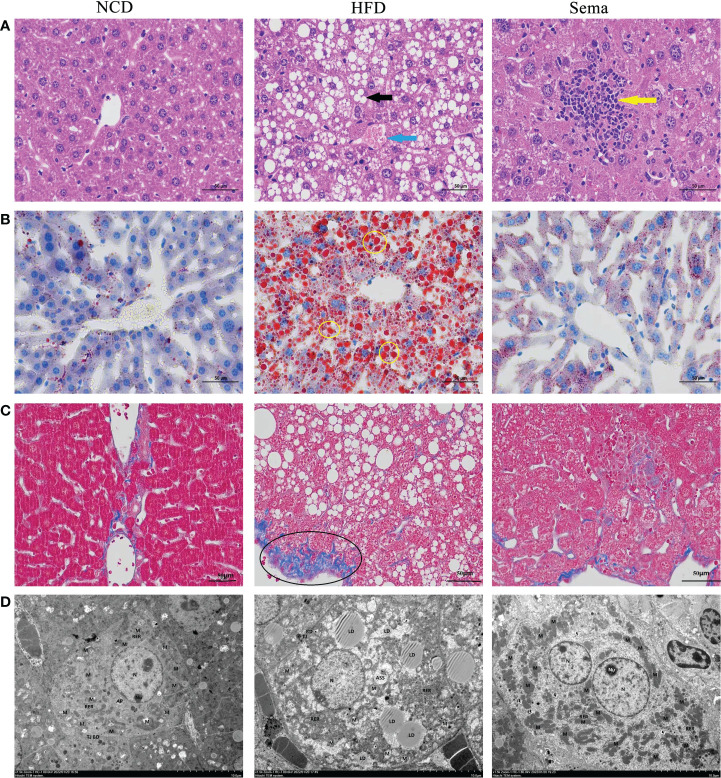
Effects of semaglutide on liver histology changes in NAFLD model. **(A)** Representative images of liver tissues stained with HE staining; **(B)** Representative images of lipid deposition stained with Oil Red O; **(C)** Representative images of liver fibrosis stained with Masson; **(D)** TEM images showed the mitochondria, mitochondrial microautophagy structure and ribosomes; NAFLD, non-alcoholic fatty liver disease.

HE staining showed that liver sections in NCD group had normal structures. Liver cells were plump and regularly arranged without obvious pathological changes in NCD group. In the liver tissue of HFD group, a large area of diffuse hepatocyte steatosis were seen, with a large number of round vacuoles (black arrows) of different sizes around the nucleus. More common hepatocyte watery degeneration, swollen cells, cytoplasmic loose (blue arrows), pale staining and focal lymphocytic infiltration were observed. After administration of semaglutide, the lesions including hepatocyte steatosis, ballooning degeneration and lymphoid foci (yellow arrows) were significantly improved (**Figure 4A**). Additionally, no abnormality in hepatocyte morphology and no lipid droplet were formatted in NCD group. Oil red O staining of liver tissue stated that the lipid deposition in the HFD group was aggravated, whereas semaglutide treatment remarkably reduced the percentage of lipid droplet (yellow circle) area (P<0.05) (**Figure 4B**). Masson staining was utilized to reflect the degree of hepatic fibrosis, and this study indicated that the poor percentage of collagen fibrin (black circle) in HFD group. And it was decreased after semaglutide treatment (**Figure 4C**). Because the time of modeling was too short, fibrosis in the liver was not obvious, so fibrosis was not obvious after the administration of semaglutide. Furthermore, TEM presented that the mitochondria in HFD group were slightly swollen, the mitochondrial cristae were partially broken and reduced, the intramembrane matrix was partially dissolved, and the mitochondrial microautophagy structure and ribosomes was visible. Consistent with the preservation of hepatic structures, semaglutide ameliorated the mitochondrial structure with less swollen and mitochondrial cristae with relatively more organized compared to NAFLD mice (**Figure 4D**). Taken together, those results supported the protecting role of semaglutide against NAFLD *via* reducing hepatocyte steatosis, lipid deposition, and mitochondrial damage.

### Effects of semaglutide on liver metabolomics in HFD-induced NAFLD model

**Figure 5:**
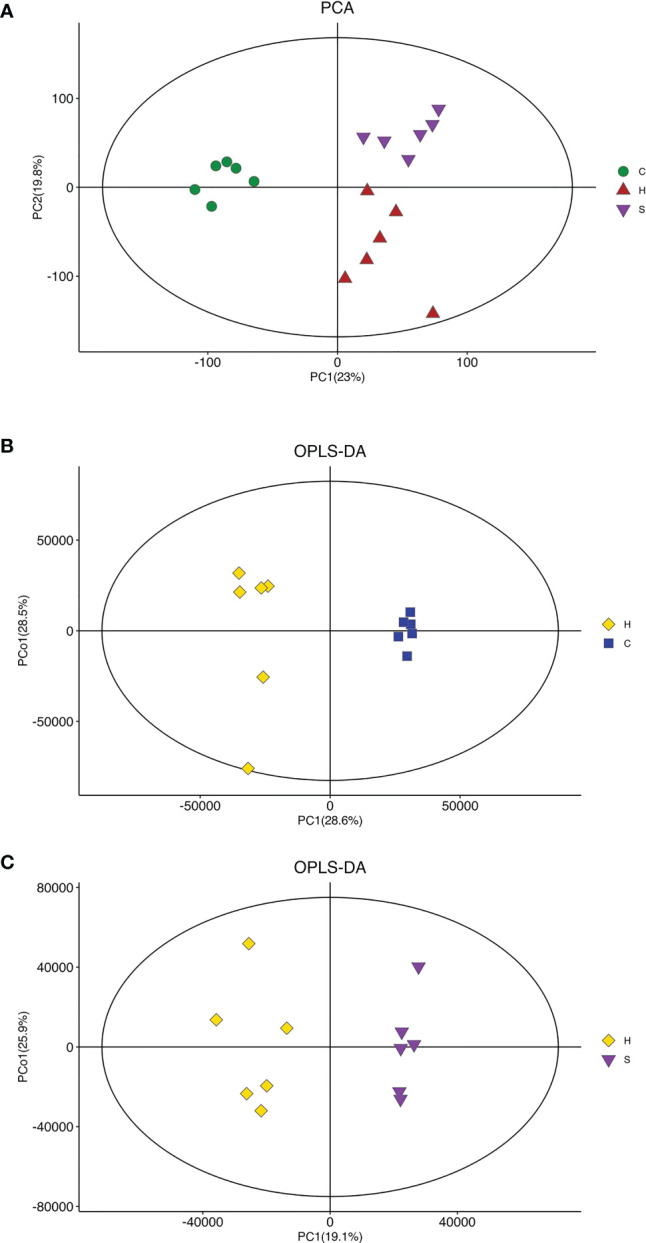
Effects of semaglutide on liver metabolomics in HFD-induced NAFLD model. **(A)** PCA was utilized to analyze the metabolites from NFD and HFD-fed rats with and without semaglutide treatment for 24 weeks; **(B, C)** OPLS-DA was utilized to analyze the metabolites between NCD and HFD group or HFD and HFD+sema group; PLS-DA, partial least squares discriminant analysis; OPLS-DA, orthogonal partial least squares discriminant analysis; NAFLD, non-alcoholic fatty liver disease.

To understand the action of semaglutide on the metabolic profile alteration, we further conducted the LC-MS of liver tissues from mice. First, The PCA score plot indicated that there was a clear trend of separation between groups, indicating significant metabolic and relatively stable system (**Figure 5A**). OPLS-DA score plot showed all groups were distinguished clearly. Additionally, the triangle representing the HFD+sema group moved to the dot representing the NCD group, indicating that semaglutide significantly modulated metabolic status of HFD group. OPLS-DA score plot displayed that manifest separation between NCD and HFD group or HFD and HFD+sema group (**Figures 5B, C**). All these results showed that the LC-MS system was with good reproducibility and stability.

**Figure 6:**
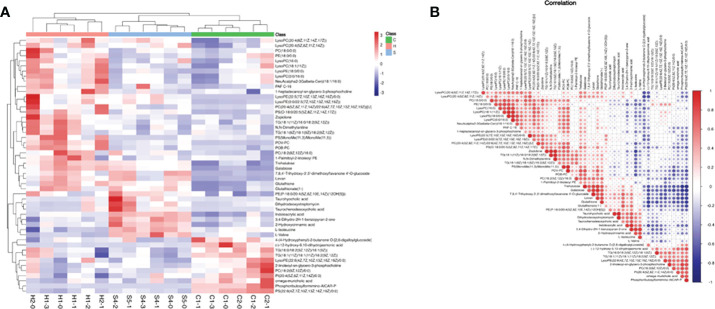
**(A)** Heatmap analysis of differential metabolites with VIP of OPLS-DA greater than 1, and P-value of Student’s test less than 0.05; VIP, variable important in projection; OPLS-DA, orthogonal partial least squares discriminant analysis. **(B)** Heatmap Pearson’s correlation analysis was conducted to evaluate the correlation of differential metabolites. Some metabolites were negatively related (blue), and some were positively related (red).

Based on the screening criteria with VIP greater than 1 and P-value less than 0.05, we found that differential metabolites mainly included amino acids, fatty acids, carbohydrates, nitrogenous compounds, and cholines, as shown in Heatmap analysis (**Figure 6A**). Heatmap Pearson’s correlation analysis was conducted to evaluate the correlation of differential metabolites (**Figure 6B**). The red and blue colors in the plot represented the up-regulated and down-regulated correlation intensity. According to the correlation coefficient, cluster analysis suggested the interaction among some metabolites.

**Figure 7:**
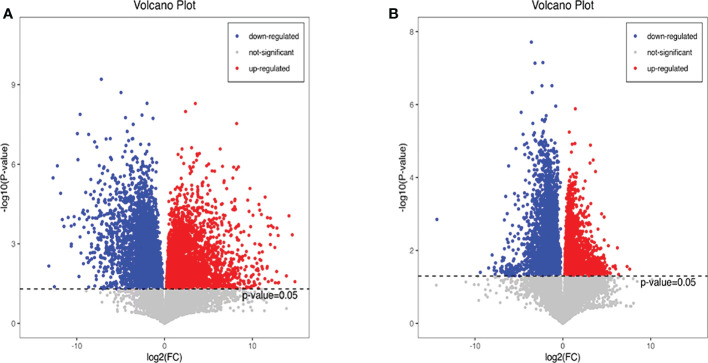
Differential metabolites with FC greater than 1 and P-value less than 0.05 were shown. The volcano plots **(A, B)** of differential metabolites in the mice of NCD vs HFD and HFD vs HFD+Sema groups; FC, fold change; NCD, normal diet; HFD, high-fat diet; Sema, semaglutide.

Additionally, we also adopted the criteria with FC greater than 1 and P-value less than 0.05, and it turned out that 19 differential metabolites were up-regulated and 13 differential metabolites were down-regulated in the HFD group compared with NFD group (**Figure 7A**; **Table 1**). However, semaglutide treatment down-regulated 10 metabolites and upregulated 4 metabolites (**Figure 7B**; **Table 2**). The results showed that 6 up-regulated metabolites in the HFD group were altered by semaglutide treatment including L-Histidinol, Arachidonic acid, Glutathione, LysoPC(16:0), TG(16:0/16:1(9Z)/18:1(9Z) and 4,5-LTA4. On the other hand, 2 down-regulated metabolites in HFD group were also restored by semaglutide treatment including L-Isoleucine and Methacholine **(** **Table 3**).

**Table 1:** The DEPs between HFD/NCD (FC value>1, upregulated proteins, FC value<1, downregulated proteins).

KEGG	Metabolites	Annotation	FC value	P value
C00860	L-Histidinol	Histidine metabolism	1.18894	0.034
C00103	Glucose 1-phosphate	Galactose metabolism	1.68831	0.01
C00243	Alpha-Lactose	Galactose metabolism	10.3004	0.002
C00984	D-Galactose	Galactose metabolism	3.17289	0.0002
C01613	Stachyose	Galactose metabolism	66.0569	0.0002
C00219	Arachidonic acid	Arachidonic acid metabolism	1.80676	0.005
C00208	D-Maltose	Carbohydrate digestion and absorption	20.8401	0.004
C01355	Levan	Carbohydrate digestion and absorption	20.6935	0.0002
C00051	Glutathione	ABC transporters	5.50334	0.0005
C00669	gamma-Glutamylcysteine	Glutathione metabolism	7.08569	0.0003
C00670	Glycerophosphocholine	Glycerophospholipid metabolism	3.57497	0.00015
C04230	LysoPC(16:0)	Glycerophospholipid metabolism	1.75061	0.0157
C04230	LysoPC(20:4(8Z,11Z,14Z,17Z)	Glycerophospholipid metabolism	1.87344	0.01289
C00064	L-Glutamine	D-Glutamine and D-glutamate metabolism	9.0535	0.0277
C00819	D-Glutamine	D-Glutamine and D-glutamate metabolism	13.8482	0.02468
C00721	Dextrin	Starch and sucrose metabolism	17.2959	0.00171
C00422	TG(16:0/16:1(9Z)/18:1(9Z)	Insulin resistance	1.6681	0.02452
C00043	Uridine diphosphate-N-acetylglucosamine	Insulin resistance	3.31479	0.04066
C02645	4,5-LTA4		4.88241	0.0392
C00439	Formimino-L-glutamic acid	Histidine metabolism	0.03011	0.00682
C02741	Phosphoribosyl-AMP	Histidine metabolism	0.02699	0.00181
C04896	PhosphoribosylformiminoAICAR-phosphate	Histidine metabolism	0.00694	0.00331
C04916	Phosphoribulosylformimino-AICAR-P	Histidine metabolism	0.02819	0.00011
C05570	Ergothioneine	Histidine metabolism	0.00216	0.00012
C05575	L-Histidine trimethylbetaine	Histidine metabolism	0.01523	0.00054
C05960	15-keto-PGF2alpha	Arachidonic acid metabolism	0.0399	0.00021
C05964	11-dehydro-TXB2	Arachidonic acid metabolism	0.39868	0.00192
C14748	20-HETE	Arachidonic acid metabolism|	0.3891	0.00035
C14749	19-HETE	Arachidonic acid metabolism|	0.1698	0.00013
C00004	NADH	Aldosterone synthesis and secretion	0.12352	0.00952
C00624	N-Acetyl-L-glutamic acid	Arginine biosynthesis	0.45795	0.01823
C01227	DHA	Steroid hormone biosynthesis	0.22734	0.00024
C07471	Methacholine		0.13864	0.00295

**Table 2:** The DEPs between HFD/Semaglutide(FC value>1, upregulated proteins, FC value<1, downregulated proteins).

KEGG	Metabolites	Annotation	FC value	P value
C00422	TG(16:0/18:1(9Z)/18:2(9Z,12Z)	Regulation of lipolysis in adipocytes	1.40119	0.02682
C00696	Prostaglandin D2	Neuroactive ligand-receptor interaction	3.9372	0.04731
C00245	Taurine	Neuroactive ligand-receptor interaction	2.253	0.00295
C00079	L-Phenylalanine	Aminoacyl-tRNA biosynthesis	0.532	0.00072
C00082	L-Tyrosine	Aminoacyl-tRNA biosynthesis	0.52658	0.00017
C00148	L-Proline	Aminoacyl-tRNA biosynthesis	0.74646	0.0108
C00183	L-Valine	Aminoacyl-tRNA biosynthesis	0.45524	0.00679
C00407	L-Isoleucine	Aminoacyl-tRNA biosynthesis	0.70402	0.00783
C00020	Adenosine monophosphate	Regulation of lipolysis in adipocytes	0.55494	0.00972
C01772	2-Hydroxycinnamic acid	Phenylalanine metabolism	0.62917	0.00091
C00077	L-Ornithine	Arginine biosynthesis	0.73271	0.0135
C00624	N-Acetyl-L-glutamic acid	Arginine biosynthesis	0.58908	0.00803
C07471	Methacholine		0.3277	0.00881

**Table 3:** The up-regulated and down-regulated metabolites in the HFD group were altered by semaglutide.

KEGG	Metabolites	Average(H)	Average(C)	Average(S)
C00860	L-Histidinol↑	18153811	15268907.04	15139503
C00219	Arachidonic acid↑	998326.112	552549.249	57032.5321
C00051	Glutathione↑	16980724.2	3085533.34	603878.8711
C04230	LysoPC(16:0) ↑	84352861.5	48184770.88	55474026.6
C00422	TG(16:0/16:1(9Z)/18:1(9Z) ↑	2217434.67	1329316.561	1381633.49
C02645	4,5-LTA4↑	474530.935	97192.03688	57098.8542
C00407	L-Isoleucine↓	13394228.1	15857001.7	19025355.7
C07471	Methacholine↓	370441.79	2671875.99	1130433.9

**Figure 8:**
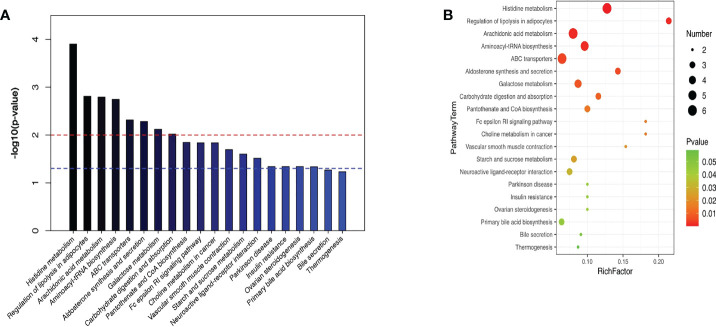
KEGG enrichment analysis stated the top ten hepatic pathways *via* bubble mapping. **(A)** A histogram **(B)** bubble mapping, KEGG, Kyoto Encyclopedia of Genes and Genomes.

KEGG enrichment analysis stated that the principal differentially metabolites following semaglutide treatment were involved in metabolic pathways of liver. The top ten hepatic pathways included the histidine metabolism, regulation of lipolysis in adipocytes, arachidonic acid metabolism, aminoacyl-tRNA biosynthesis, ABC transporters, aldosterone synthesis and secretion, galactose metabolism, carbohydrate digestion and absorption, choline metabolism in cancerhe, and insulin resistance (**Figure 8**).

## Discussion

The HFD mouse model was prone to liver steatosis and can replicate most of the metabolic characteristics of human NAFLD. Thus, this model can be used to investigate NAFLD and related metabolic syndromes (12). In the present study, body weight, and liver weight were significantly higher in the HFD group than in the control group after 12 weeks of HFD feeding. There were studies using the same or similar dietary composition and the same duration to successfully induce steatosis, inflammation and no fibrosis (12, 13). In addition, abnormal serum transaminases, serum and hepatic lipid levels. And NAFLD histology features steatosis, inflammation, and ballooning. These characteristics indicated that the rat NAFLD model has been successfully constructed.

There was a study reported that NAFLD exhibits elevated levels of SOD and oxidative stress, which lead to changes in mitochondrial function (14). Consistent with the published research results, we detected the increase of SOD, a marker of oxidative stress in the blood. Under the electron microscope, mild mitochondrial swelling, increased volume, local fragmentation and reduction of cristae, and local dissolution of the intramembrane matrix, and mitochondrial micro-autonomy can be seen in the visual field. Large areas of diffuse hepatocyte steatosis with numerous round vacuoles of various sizes surrounding the nucleus were seen in HFD liver tissue. Watery degeneration of hepatocytes and focal infiltration of lymphocytes are more common. Semaglutide can significantly improve the pathological changes of hepatocyte steatosis, ballooning degeneration and lymphoid foci. Semaglutide relieves lipid accumulation in the liver. The HFD group had significant collagen deposition, and semaglutide exhibited significant anti-fibrotic activity, greatly reducing the percentage of collagen area. All pathological results confirmed that semaglutide alleviated hepatic lipid accumulation, inflammation, and fibrosis morphologically, and led to changes in mitochondrial structure.

Wang et al. showed that FA and its metabolites are detrimental factors in the development of NAFLD (1). The major pathophysiological mechanisms of NAFLD involve increased FA accumulation and decreased mitochondrial FA oxidation. It leads to liver cell lipid metabolism dysfunction and lipid accumulation as well as hepatocyte damage caused by activation of tumor necrosis factor-α and stimulation of reactive oxygen species (ROS) production (1). Under normal physiological conditions, the synthesis and degradation of free fatty acids, triglycerides and total cholesterol in the body are in a dynamic equilibrium (15). Hepatic steatosis is caused by an imbalance in hepatic lipid accumulation. Due to the breakdown of visceral adipose tissue fat, the synthesis of new fat in the liver, the increase in the consumption of a high-calorie high-fat diet, and the reduction of hepatic mitochondrial β-oxidation. Ultimately leads to increased production and accumulation of plasma free fatty acids, low-density lipoproteins, and triglycerides (16).

L-Histidinol is an enzyme that regulates histidine biosynthesis. Studies have reported that excessive intake of histidine can cause hepatic steatosis (17). May be due to an imbalance between lipid transport in the liver and free fatty acid uptake into hepatocytes. LysoPC(16:0) is a phospholipid whose elevated levels increase the risk of liver injury and oxidative stress, and phospholipid metabolism is closely related to the pathogenesis of NAFLD. The concentration of LysoPC(16:0) metabolite was higher in patients with different degrees of hepatic fat accumulation (18). L-Isoleucine is a Branched-chain amino acids (BCAA). Previous studies have reported that BCAAs can promote fatty acid β-oxidation and ketone body production (19). β-oxidation is thought to be the main mechanism of hepatic lipid reduction and is involved in the upregulation of peroxisome and mitochondrial genes (20). Methacholine is an acetylcholine receptor agonist. Studies have found that patients with long-term parenteral nutrition lack of choline, after choline supplementation, there will be a reversal of hepatic steatosis and a decrease in serum transaminases. Pathological changes reappeared after choline supplementation was discontinued (21, 22). L-Isoleucine and Methacholine were significantly down-regulated in HFD group and NFD group, but were significantly up-regulated after semaglutide intervention. Semaglutide reduces lipid droplet formation, inhibits adipose tissue lipolysis and regulates phospholipid homeostasis by downregulating L-Histidinol, LysoPC(16:0) and TG. Consistent with our results in liver histology, by increasing L-Isoleucine and Methacholine levels to promote fatty acid β-oxidation and ketone body production, changes in metabolites alleviated hepatic lipid accumulation.

The pro-inflammatory cytokines (IL-6 and TNF-α) released during the pathophysiology of NAFLD are mainly derived from macrophages (23). IL-6 is both a pro- and an anti-inflammatory factor in many biological processes. TNF-α is a central link in the inflammatory cascade and can promote the release of other pro-inflammatory substances, thereby accelerating the inflammatory process (24). IL-6 and TNF-α can directly reflect the degree of inflammatory response in the body (25). Consistent with our experimental results, semaglutide inhibited the up-regulation of pro-inflammatory factors (TNF-α, IL-6, IL-1β, MDA). Semaglutide reduced but did not fully normalize hepatic inflammation. This may be related to the dose and timing of semaglutide.

Arachidonic acid, a polyunsaturated fatty acid metabolized by cyclooxygenase and lipoxygenase pathways, can mediate inflammation and participate in the development of NAFLD. The study of Hall (26) et al. showed that phospholipid membrane is remodeled in late NAFLD stage, and arachidonic acid is released from phospholipid membrane, thereby aggravating cell damage and inflammatory response. Arachidonic acid can be converted into LTA4 (5,6-epoxide of arachidonic acid), and 4,5-LTA4 is a precursor of leukotrienes. Leukotrienes are a class of classical pro-inflammatory lipid metabolism intermediates that trigger and amplify inflammatory responses (27). Inflammation is an important factor leading to liver damage. This study is consistent with previous studies: arachidonic acid and 4,5- LTA 4 are significantly up-regulated in HFD group, while the metabolites prostaglandin D2 and 4,5-LTA4 of arachidonic acid are significantly down regulated after semaglutide treatment. Therefore, semaglutide may reduce the inflammatory response of NAFLD and liver cell damage in mice by down regulating the expression of inflammatory factors arachidonic acid and 4,5- LTA 4. A limitation of this study is the lack of further confirmation by *in vitro* experiments, which should be further evaluated in future studies.

## Conclusion

Semaglutide can reduce blood glucose level and liver fat accumulation and play an anti-inflammatory role in advanced NAFLD that due to the effects of HFD.

## Data availability statement

The original contributions presented in the study are included in the article. Further inquiries can be directed to the corresponding author.

## Ethics statement

The animal study was reviewed and approved by The Animal Ethics Committee of Hebei General Hospital.

## Author contributions

SN and SC conceived and designed the study. SN, QR, LY, and XinC provided materials and samples. LY, QR, SN, XP, XiaC, HZ and ZL collected and assembled the data. SN and SC analyzed and interpreted the data. All authors contributed to the article and approved the submitted version.
